# Vaccine Hesitancy in the Age of Coronavirus and Fake News: Analysis of Journalistic Sources in the Spanish Quality Press

**DOI:** 10.3390/ijerph17218136

**Published:** 2020-11-04

**Authors:** Daniel Catalan-Matamoros, Carlos Elías

**Affiliations:** 1 Department of Communication Studies, University Carlos III of Madrid, 28903 Madrid, Spain; celias@hum.uc3m.es; 2 Health Research Centre, University of Almeria, 04120 Almeria, Spain; 3 Research Group for Analysis and Anticipation Journalism, University of Nebrija, 20240 Madrid, Spain

**Keywords:** content analysis, media, newspaper, public health, sources, journalism, vaccine

## Abstract

The study of the quality press and the use of sources is relevant to understand the role of journalists in scientific controversies. The objective was to examine media sourcing patterns, using the case of vaccines as a backdrop. Articles were retrieved from the national quality press in Spain. Content analysis was undertaken on the sources and on other variables such as tone, frames and journalistic genre. The software myNews and NVivo were used for data collection and coding, while SPSS and Excel were used for statistical analysis. Findings indicate that sources related to the government, professional associations and scientific companies are the most frequently used, confirming the central role of government institutions as journalistic sources. These were followed by university scientists, scientific journals and clinicians. On the other hand, NGOs and patients groups were included in fewer than 5% of the articles. More than 30% included none or just one source expressing unbalanced perspectives. Frequent use of certain source types, particularly governmental, may indicate state structures of power. The study provides a better understanding of journalistic routines in the coverage of vaccines, including fresh perspectives in the current COVID-19 pandemic.

## 1. Introduction

In order to improve society, accurate science-related information is needed. Aside from primary and secondary school textbooks, the media are considered the most used source of scientific information by the population and, for the majority, the unique source for science-related topics [1]. As the lay public does not maintain fluent contacts with scientists or healthcare professionals [2], the media are crucial in keeping the public informed about scientific issues. The information that they receive from the mass media has the potential to shape the public’s perception of issues and events, opinions, attitudes and even behaviours [3].

In our expanding media universe, with increasing coverage of health and science issues [4], sourcing is considered an important task in journalism practice and is a key component in story construction, which provides journalists with story content and context [5]. Needless to say that, in the current COVID-19 infodemic, with the increasing spread of science and health mis/disinformation, careful news production is required by journalists in order to avoid or minimise the spread of fake news to the public [6,7]. Journalists have the power to select their sources and are often challenged to find interesting and trustworthy voices beyond their networks and socio-cultural environments. The concepts of accuracy and neutrality are treasured in any type of journalism, and identifying credible sources to help tell a story and source verification become vital [8]. Research studies have found that journalists are more and more dependent on sources, a trend which is even stronger in science journalism [9]. In journalism research, the field of journalistic sources is inseparably related to agenda setting (what) and framing (how) [10]. Thus, the analysis of sources is central to increasing our understanding of news content and production [11]. Although all authors agree that journalism is driven by sources, research on sourcing is still quite limited [12], particularly in relation to specialised journalism. Previous research of this kind has been undertaken especially in the United States [11]. Therefore, it is necessary to analyse the different roles of the mass media in spreading information to the public in other world regions with different cultures and political systems. Using the case of vaccines as a backdrop, and by conducting content analysis of the quality press, our research aims to analyse journalistic sourcing patterns. The World Health Organization (WHO, Geneva, Switzerland) ranked vaccine hesitancy as one of the most important 10 health threats in the world [13].

In this study, we want to identify the particular use of sources which may be crucial in the population’s understanding of risks about vaccines; indeed, these actors may describe a public health perspective which has not been clearly detailed by previous research. As content analysis is considered an established method of measuring sources’ characteristics [14], the analysis will, firstly, explore which sources are commonly used in the coverage of vaccines, and, secondly, discover the different characteristics of sourcing.

### 1.1. Literature Review

#### 1.1.1. The Journalistic Sources

Some types of journalists, such as sports journalists and war correspondents, can bring to the public first-person information regarding what is happening. Other journalists have to cover events that take place outside of their immediate experience, and must obtain the necessary information from sources. The use of expert sources and quotes allows journalists to appeal to others’ voices to frame stories in certain directions [5]. Some relevant functions of journalistic sources have been identified [15,16]: (1) presenting how to verify the news sources; (2) increasing reliability; (3) avoiding ambiguity; (4) showing various points of view; and, finally, (5) protecting against accusations of bias.

In daily journalistic work, sources are a key element of news making. There is a significant source dependence during the story building process, although this is normally less relevant in the advanced stages of information search [17]. Previous research has analysed sources in specific settings [12] and in specific cases or topics [5,9,18]. These analyses of sources provide important insights into the journalistic routine of news production [9].

Source pluralism is dependent on a variety of elements and can therefore differ in unlikely situations [12]. A broad analysis of sourcing patterns is thus key to understanding how news coverage is built and for confirming whether the public can receive enough knowledge for well-informed decisions from the mass media. Source selection criteria depend on specific determinants [12]. The first determinant is the journalists’ trust in main source characteristics, which includes the level of knowledge about the source and its credibility. Second is the sources’ communicative activities, such as whether they take journalistic practices into consideration by being available to be interviewed. Third, the source selection criteria also depend on the conversation between the expert and the journalist; for example, journalists more often trust expert sources that have been assembled in the past. However, before journalists select their sources, the news is shaped by their particular ideas or by the editorial type of discourse. In this regard, the following quote (Schneider 2012, 9) represents how journalists may use interviews to fill spaces in story building:

As someone who has on occasion been interviewed for news reports, I have been frustrated by journalists’ attempts to get me to provide a particular answer for an item they already have in mind, rather than being willing to hear my thoughts about what the story is or could be.

Source selection is also influenced by the well-known journalistic concept of balance [19]. Many issues are covered by the ethical code of impartiality, and it has been argued that the most important way to conduct a sourcing study is by analysing how news coverage is properly balanced on the overall spectrum of different views or perspectives. Moreover, another factor that may influence source selection is expressed by agenda-setting theory, which explains how journalists select sources according to the particular agenda that the media organisation wishes to develop [20].

#### 1.1.2. Sources in Science Journalism

Journalists who are specialised in science, known as science journalists, think of themselves as bridges between the lay public and experts or scientists [20]. The emergence of scientific and health communication is characterised by the steady amount of research articles published and by increasing public concern over scientific issues, such as the global spread of infections and global warming [21]. However, there are some obstacles which journalists face: tight deadlines, lack of reliable sources, increased commercialisation and budget and staff cuts [22].

As is acknowledged, the press environment is where scientists meet the public [23] and journalists report, but do not make, the news. News must be supported by scientific knowledge such as data and facts. Journalists frequently mention a variety of sources, but they believe that scientists are considerably more reliable than other source types [20]. Journalists invite scientists as experts to describe, illuminate and explain the stories, and also to increase reliability [22]. This is the reason why expertise is sought after and seen as a crucial element when covering specialised issues. Indeed, one of the main job components of journalists is looking for suitable expert sources. Although science journalists aim to develop a “symbiotic relationship” with scientists [24], achieving this relationship is not easy, since scientists and journalists have different objectives and make decisions based on different principles. As an example, the concept of “publication timing” is very different between journalists and scientists. While journalists need to prepare publications in days or sometimes in hours, the publication process of scientists lasts much longer (several months or even years). These differences have caused some conflicts in this relationship. Some scientists have reported positive experiences when working with journalists, although others, especially those fearful of being misquoted, have reported that journalism is poorly transparent, imprecise and does not properly reference scientific evidence [25,26].

A science journalist not only informs the public about the latest scientific developments and findings, but is also a watchdog to critically identify scientific controversies [26]. A journalist does not work for scientists but for the lay public. In addition to the use of scientists as sources, it is known that many different sources (e.g., politicians, companies, patients) can also be key in a story about a scientific issue. As such, through our paper, we try to explore and describe this phenomenon and to provide a structured initial analysis to improve our general understanding of sourcing in specialised journalism.

#### 1.1.3. The Case of Vaccines and the Media

Vaccines constitute one of the main advances in history, as they have eliminated many infectious diseases [27]. The decline in communicable diseases thanks to vaccines has been one of the greatest accomplishments in the preventive field, but this measure needs to be followed by the public [28]. Currently, there is an antivaccine lobby in society which influences vaccine adherence. In the European region, vaccination is falling to a very risky level and vaccine preventable diseases are again being found across Europe [29].

The theme of vaccines has received large media coverage, mainly due to safety issues and controversies. The media are believed to be an important means of spreading vaccine-related information, improving awareness and preparing the public for well-informed decisions about health [30,31]. Due to this media attention, scholars have analysed media contents about vaccines. A systematic review shed light on the nature of communication about vaccines in the traditional media [32]. One important finding was that the majority of research had been conducted in the United States and that therefore there is a research gap about vaccines communication in other regions of the world. Our paper will help to advance this research field by analysing media coverage of vaccines in the national media of Spain, which is ranked as the fifth most populous nation in the European Union.

#### 1.1.4. Vaccines in the Age of Coronavirus and Fake News

COVID-19 is the first pandemic in the global era of widespread mobile device-supported social media, big data and AI. This has made it so that misinformation is rapidly and more thoroughly spread throughout the public, to the extent that the WHO coined the word “infodemic” to describe an overabundance of information and the rapid spread of misleading or fabricated news, images, and videos [33]. COVID-19 has led to a parallel pandemic of disinformation that directly impacts lives and livelihoods around the world.

Antivaccine groups are becoming stronger and they are expected to become ever more active once COVID-19 vaccination starts. Indeed, since the beginning of the coronavirus pandemic, there have been many vaccine-related conspiracy theories circulating, such as that the virus was created by pharmaceutical companies to sell a vaccine or that Bill Gates is creating a tracking device to be implanted in a COVID-19 vaccine.

Health communication experts say they need to start to lay the groundwork for vaccine acceptance now, because the flood of misinformation from antivaccine activists has already surged [34]. Recent polls have found as few as 50% of people in the United States are committed to receiving a COVID-19 vaccine, with another quarter wavering. Some of the communities most at risk from the virus are also the most leery: among Black people, who account for nearly one-quarter of U.S. COVID-19 deaths, 40% said they would not get a vaccine in a mid-May poll by the Associated Press and the University of Chicago. In France, a recent survey found that 26% of the population would not get a coronavirus vaccine [34].

### 1.2. Research Questions

As the aim of the current study is to obtain a more nuanced understanding of the sourcing patterns in specialised journalism using the case of vaccines as a backdrop, we aim to provide answers to the following research questions.

First, an exhaustive search concerning sourcing in news articles on vaccines yielded no studies which were directly relevant to the current research. Thus, the issue of whether there is a diversity of sources when covering the specialised topic of vaccines in the news is a very pertinent research question. Sources can be identified and grouped in different ways according to the literature review, for instance, into political and non-political sources [16]. The second question tries to identify whether there is a balance among the variety of sources, i.e., scientists, politicians, patients, scientific journals, etc. This will aid the understanding of general sourcing patterns in specialised journalism.

*RQ1. Which sources are used in Spanish dailies about vaccines or vaccination?*

*RQ2. Do journalists follow the principle of balance when using sources, and are some sources more used than others?*

The third research question will focus on the number of sources cited in a news story. The aim is to identify key patterns related to the number of sources that are used by journalists. This analysis has become even more pertinent as news companies are now more profit-oriented, meaning that the burden to raise production has substantially intensified [11,35]. Thus, time for the production of a news article is more limited and there is the potential risk of a reduced amount of verification or fact-checking.

Tiffen et al. (2014, 5) stated that a story based on a single source allows that source’s view of events to be carried unchallenged, and reflects a passive orientation. In contrast, the use of a variety of sources indicates an active news media orientation, allowing checks and enabling balanced views [26,36]. The following phase is analysing the relation of sources to other characteristics, such as the tone of the article, frame, type of vaccine and length of the article. Based on the above, we have established that the use of none or one single source in a news article is considered an inappropriate journalistic practice, while the use of two or more sources could be a positive practice, as previously suggested by Schneider (2012). Therefore, the following relevant research question will be examined:

*RQ3. How many sources do journalists use when covering vaccine-related topics and what is the number of sources related to the tone, journalistic genre, frame and length of the article?*

## 2. Materials and Methods

We conducted content analysis [37] of journalistic contents in a selection of major national newspapers in Spain. We examined source choices for stories published during a 5-year period, from 2012 to 2017, seeking to identify the source use patterns in the media coverage of topics about immunisation.

We used myNews as the online database to search in the two paid general newspapers with the highest circulation rates in Spain according to the General Media Study [38]. We were interested in analysing the coverage in the “quality press”. The International Encyclopedia of Communication [39] notes that there is an unwritten consensus in defining “quality press” as newspapers and magazines that: (1) are addressed to the “intelligentsia”, that is, the elites and the decision-makers of a country; (2) are distributed nationally, never regionally; and (3) provide broad and in-depth news coverage, contextualising the information by providing background [40]. It also indicates that the “quality press” frequently cooperate with each other. At the moment, there is concern in many sectors about the dissemination of disinformation among the media, and emerging concepts such as “fake news”, “alternative facts” and “post-truth” are increasingly analysed in the academy [41,42,43]. The quality press could be a useful tool in fighting against the dissemination of fake news [43], especially because of the way the quality press selects the sources of its news (based on their expertise). In this sense, both newspapers—El Mundo and El País—can be considered “quality press” in Spain.

The search in the databases used the following keywords [vacuna* OR inmuniza*], which needed to be located either in the headlines or subheadlines in order to obtain relevant articles about vaccines or vaccination. Any type of article was included such as features, news articles and opinion articles. While the word “article” is used throughout this study, it should be recognised that this includes the other article types just mentioned. We believe that newspapers still remain an important traditional medium and are considered reliable sources by the public [44].

We imported the articles into QSR NVivo 11 plus to perform the categorisation of code frequencies. One researcher performed the analysis following a standardised method to collect the journalistic genre (news article, feature, etc.), the type of vaccine that was mentioned, amount of words in the article, tone and frames through a deductive method [45]: human interest, conflict, mortality, economic consequences and responsibility. Each article was read and re-read, and the focus was on looking for keywords, metaphors, phrases and sentences. In addition, the main theme of each article was collected to have a data context.

The sources were classified, according to the affiliation of the individual, in the following categories: “government scientific organisations”, such as the National Regulatory Medicines Agency and the National Health Institute Carlos III; “government organisations”, such as the Ministry of Health (minister, state health secretary, etc.), regional health administrations and international organisations; “scientific companies”, including the pharmaceutical and health technology sectors; “university scientists”, including researchers affiliated with any university or research centre; “clinicians”, including any health professional working at any healthcare centre; “scientific journals”, including any scientific peer-reviewed publication; “media”, such as a press agency or a media channel; professional associations, including any organisations composed of health professionals as members, such as the Spanish Association of Pediatrics (AEP) and the Spanish Society of Public Health and Health Administration (SESPAS, for its Spanish acronym); “consumer groups”, including representatives from patients or users’ associations; and “NGOs”, including any non-governmental organisation used as a source. The category “other” was used when a source was not able to be included in any of these categories. In addition, the sources have been grouped into two large categories in the present study: scientific and non-scientific sources. Within each category, we have identified a number of sub-categories. Under scientific sources, we have the following sources: “government scientific organisations”, “professional organisations”, “scientific companies”, “university scientists”, “clinicians” and “scientific journals”. Moreover, under the non-scientific sources, there are: “media”, “government organisations”, “NGOs”, “consumer group representatives” and “others”.

For coding reliability, one researcher (DCM) performed a first coding round, while a second round was performed by another researcher (CSO). For any discrepancy, we were advised by a third researcher (CPS) when necessary. For data analysis, we used Excel (Microsoft Corporation, Redmond, WA, USA) and SPSS 24th edition (SPSS Institute Inc., Chicago, IL, USA). Next, we followed the methodology conducted in a previous similar study [20] as follows: (a) chi-square and goodness of fit analyses were employed to determine whether the category distribution significantly differed from an expected even distribution; (b) *t*-test analyses were conducted when possible; and (c) bivariate correlation and the Spearman rho test of statistical significance were also performed, as the data under analysis were nonparametric.

## 3. Results

The search yielded 159 articles. Of these, twenty-eight were excluded as duplicates. We analysed 131 journalistic contents. In El Pais, there were 75 articles, and in El Mundo, there were 56 articles, with no differences (*p* = 0.97). Table 1 shows the general features for the sample.

With regard to the analysis of sources, Table 2 displays descriptive statistics for the variety of sources. In total, 374 sources were found among the 131 articles, with a mean of 2.8 sources per article. “Government scientific organisations” were the most frequent type of source (25.4%, *n* = 95), followed by “professional associations” (16.5%, *n* = 62), political leaders representing “government organisations” (15.2%, *n* = 57) and “scientific companies” (10.4%, *n* = 39). The other types of sources, although with a lower frequency, were “university scientists”, “scientific journals”, “clinicians”, “NGOs”, “media” and “consumer groups”. Results from a chi-square analysis revealed that the distribution of sources was not significantly different, χ^2^ (df = 8) = 1.27, *p* = 0.996.

In addition, Table 2 shows the number of sources used in each of the articles (i.e., one source, two sources, etc.). Twelve articles (9.2%) did not reference any source, and 30 articles (22.9%) referenced only one source, this being the most commonly used pattern in the study sample. Indeed, results from a chi-square analysis showed that the distribution of the number of sources in the articles was significantly different, χ^2^ (df = 9) = 66.18, *p* < 0.001. Therefore, we explored the differences in type of vaccine, tone, journalistic genre, length and frames of the article in relation to the sources count, either 0–1 source or ≥2 sources per article. In relation to the tone and type of vaccine, there were no significant differences between either sourcing patterns (tone: χ^2^ = 2.18; df = 2; *p* = 0.337; type of vaccine: χ^2^ = 21.28; df = 23; *p* = 0.564). However, in relation to the journalistic genre, we found significant differences (χ^2^ = 38.78; df = 6; *p* < 0.001). Specifically, the genres “news” and “features” showed a higher frequency of articles with ≥2 sources (48 vs. 9, and 24 vs. 5, respectively), but for “short news” and “opinion articles”, we found the opposite, specifically a dominance of articles with 0–1 source (10 vs. 13, and 2 vs. 13, respectively). In relation to the frames, we also found significant differences (χ^2^ = 17.90; df = 4; *p* < 0.05). This was particularly so in the frames “responsibility” and “morality”, which showed a higher frequency of articles with 0–1 source (2 vs. 7, and 0 vs. 4, respectively). In relation to the length of the article, we also found significant differences between the two types of journalistic practice (t = −4.03; gl = 129; *p* < 0.001); the articles including ≥2 sources had a mean of 581 words, while those including 0–1 source had a mean of 346 words.

In order to obtain a broader perspective on the use of scientific sources vs. non-scientific sources, we grouped them into two categories. The first was the category “scientific sources”, which included: “government scientific organisations”, “professional associations”, “scientific companies”, “university scientists”, “scientific journals” and “clinicians”. On the other hand, the following sources were grouped under “non-scientific sources”: “government organisations”, “NGOs”, “media”, “consumers group representatives” and “others”. The results showed that the distribution of sources between the two groups was not significantly different (χ^2^ = 0.05; df = 1; *p* = 0.830). Interestingly, in a cross table, we could compare the use of scientific sources vs. non-scientific sources in each of the 131 articles included in the sample. We found that 12 (9.2%) articles did not use any source and that 11 (8.4%) articles only used non-scientific sources, meaning that the use of scientific sources was avoided in at least 23 articles (17.6%). In contrast, 49 (37.4%) articles used both types of sources (scientific and non-scientific ones), while 59 (45.0%) articles used scientific sources only, meaning that non-scientific sources were avoided in 108 articles (82.4%). These findings revealed that when journalists wrote articles without using any scientific source, all of them (100%, 23/23) included only 0–1 source. However, when journalists used scientific sources only, 56% of the articles (40 out of 71) cited ≥2 sources. Indeed, we found significant differences between the number of sources included in the article and scientific sources (χ^2^ = 101.38; df = 8; *p* < 0.001), as well as a high direct association (C = 0.661; *p* < 0.001). On the other hand, we found neither significant differences (χ^2^ = 9.89; df = 5; *p* = 0.078) nor a significant association (C = 0.265; *p* = 0.078) between the number of sources and non-scientific sources. Therefore, we might state that the use of scientific sources is directly associated with the number of sources. In other words, the more sources are consulted by the journalist, the more scientific sources are used.

While content analyses in media coverage have been predominantly descriptive [26], in order to go beyond discerning descriptive differences, we tried to determine if consistent relationships existed between the use of sources and the type of vaccine, tone, frames or length of the article. For this purpose, we created a bivariate correlation; the Spearman rho test of statistical significance was used because the data under analysis were nonparametric, as happened in the majority of variables, according to the Kolmogorov–Smirnov test [46]. As shown in Table 3, the tone was only significantly correlated with professional association sources (*p* < 0.05). Descriptive analysis showed that these sources were used more frequently for positive (*n* = 14) and neutral (*n* = 10) articles than for negative ones (*n* = 6). In relation to the journalistic genre, it was found to correlate with scientific journal sources (*p* < 0.05), as the majority of the use of journal citations was implemented in news (*n* = 17) and features (*n* = 8). In contrast, journals were rarely used in short news (*n* = 2) and opinion articles (*n* = 1), and never in other genres such as interviews or biographies. The vaccine type was correlated with professional associations sources (*p* < 0.05), which were largely used in articles about chickenpox (*n* = 10). In relation to the frames, these correlated with scientific companies (*p* < 0.001) and scientific journal (*p* < 0.01) sources. We found a higher frequency of “scientific company” and “scientific journal” sources in articles with a human interest frame (*n* = 18, *n* = 22, respectively). The length of the article, as measured in absolute number of words per article, was not correlated with any of the dependent variables. Finally, when we grouped the sources into either scientific or non-scientific categories, more significant correlations were found. Here, the journalistic genre was strongly correlated with both scientific sources (*p* < 0.001) and non-scientific sources (*p* < 0.05). Both the type of vaccines and the frame of the article were significantly correlated (*p* < 0.05) with scientific sources. The length of the article was significantly correlated with both scientific (*p* < 0.001) and non-scientific sources (*p* < 0.01).

## 4. Discussion

The data in this article provided insights on journalists’ routines when covering the specialised topic of vaccines or vaccination. We analysed the use of sources and their relation with the frames, type of vaccine, the tone and the length of articles. This study demonstrated that sources related to the government, professional associations and scientific companies are the most cited in news articles about vaccines, while it also confirms the central role of government institution sources in specialised journalism.

The data analysed for this study illustrated that the types of sources whose voices are most heard are related to “government scientific organisations” such as the National Public Health Institute (Instituto de Salud Carlos III), “professional associations” such as the Spanish Association of Pediatrics, “government organisations” where politicians’ voices are included, “scientific companies” representing the industry and “university scientists”. These findings are supported by Schudson [47,48], who stated that news is generally dominated by institutional and political sources. This could be because corporate and official sources are largely used by journalists, as they are easily available and deliver endorsed information [49]. Previous authors [50] also confirmed that in science journalism, there is a tendency to prioritise sources from governmental institutions and companies, and that people are given low priority by journalists in the process of story building. This is also aligned with our findings, as sources related to civil society, “NGOs” and “consumer groups” were fewer than 5% of the total number of sources. It has also been stated that the media in a democracy should give voice to social constituencies and currents of opinion [11]. In addition, science is limited and uncertain, meaning that it requires other types of sources to examine issues, such as sources drawn from civil society [51]. In this regard, some basic guidelines lean towards using scientific sources, although other guidelines for public/civic journalism [52] suggest to use other sources in addition to scientific experts to improve civic participation. Having other types of sources may encourage the coverage of novel issues with other, new perspectives.

Our findings indicate that “government scientific organisations” and “government organisations” represent the majority of the types of sources employed, with more than 40% of the sources used in total. However, other, more politically independent sources, such as “scientific journals” and “university scientists”, are less frequent. In science issues, the power of politics seems to be more visible than the quality of evidence-based and scientific research [8]. These findings should be put into context. In Spain, the sources located within a power structure, namely political sources, are crucial in the journalistic profession, as science journalists focus faithfully on the government side, providing a central role to politicians [53]. In our paper, we do not intend to support any specific sourcing practice model in science journalism, but to present our results and compare them with previous research. In this regard, Secko et al. [51] identified four models of how science journalism can be produced: science literacy, contextual, lay expertise and public participation. Our findings show nuances of the four models, although the most dominant one is the public participation model, which addresses policy issues related to the public understanding of science. In contrast, the science literacy model, which values expert scientific sources above others, is less frequent in our study.

Another important point is the number of sources used in the articles. The use of multiple sources has been described by different authors as a way of bringing more balance to, and better checks on, the views presented [26,36]. In contrast, the use of none or only one source has been critiqued [11], as it could cause the view of the story to be unchallenged and unbalanced. In our study, we found that each article included almost three sources on average; this figure is higher than that yielded by a recent study which found two sources on average, but in the media coverage of Belgian health news in general [10]. However, more than 30% of articles included none or just one source. The risk of this way of producing stories is that journalists might perform only as passive senders of leading sources’ perspectives. This was previously cited under the concept of “churnalism” [54], indicating that pressure on journalists to speed up or escalate the making of stories facilitates less balance and verification of diverse perspectives. In relation to the use of a low number of sources, we found significant differences between opinion articles and other journalistic genres. This could be due to the fact that there is a large number of articles smaller than 100 words and falling under the journalistic genre of opinion. In fact, 87% of opinion articles included none or just one single source. This is known as the interpretive style of writing, where journalists use strategic frames to express their opinion, explain why something happened or speculate about the future without referring to verifiable facts. Our study shows how these opinion articles express only one viewpoint and thus are “unbalanced” in terms of sourcing. Although systematic research on this phenomenon is insufficient [55] to discern whether it is best to include more sources to ensure the principle of balance, the media have received complaints from scientists for providing equal balance or weight to unfounded claims about the MMR vaccine [56]. Therefore, it is not suggested that the principle of balance be applied between sources with a high scientific evidence level and others that do not have any scientific evidence [8].

Finally, the authors believe that in the era of misinformation and fake news, journalists should be empowered to ensure the best use of sources on topics relevant for society such as vaccines. This study provides some key insights that can also be applied in the current coronavirus pandemic. Taking into account that the world is anxiously waiting for a vaccine, the antivaccine lobby is already generating mis/disinformation around COVID-19 vaccination. In this context, a recent study has found that two-thirds of British people recognised that the COVID-19 pandemic has made them appreciate journalism more than they did before the crisis, especially in its function of gathering reliable news and information [57]. We believe that improving journalistic coverage in health-related topics will make a well-informed citizenry. Based on our study, we would recommend that journalists not only filter information to stop the spread of fake news, but also that they use a variety of sources beyond governmental ones, such as consumer groups or patients’ associations, clinicians, scientific journals and scientists, especially those from universities that have shown higher degrees of neutrality when compared with those sources from scientific governmental organisations [19]. This may provide a more realistic representation of vaccine-related news, and may help journalistic contents in the media to receive better perception and recognition from the public than other contents in social media where mis/disinformation and fake news are frequent.

We also would like to acknowledge some limitations. First, some media researchers recognise that the construction of news is embedded in the journalistic culture of a particular country [58]. As the study sample is based in Spain, the results might not be applicable to other nations. Another limitation is that since we are only analysing newspapers, we cannot cover the entire media landscape with our study. However, newspapers are largely used in research, as they are good indicators for what kind of news is being broadcasted by other media formats [37]. In any case, our study offers a structured initial analysis for understanding sourcing patterns in specialised journalism. In addition, this study may offer new insights for further research, especially into how the use of journalistic sources can influence public understanding of health risks. To achieve this, future empirical research would be needed.

## 5. Conclusions

In conclusion, our findings contribute to the theoretical and practical development of journalism. Our analysis adds new knowledge to the limited understanding of the news media in their national setting and sheds light on the selection of sources. The heavier use of some sources, particularly governmental, may illuminate state structures of power. We hope that our analysis will aid the larger effort of improving journalistic professional patterns. Finally, it would be naïve to say that journalists should simply write in a different way about vaccines to improve vaccine adherence. Many elements make this goal very difficult to accomplish. An important practical measure to improve source use in coverage of vaccines could be to train and inform journalists on the variety of sources they could use, alongside the provision of some advice. This kind of training might be important, because it has been suggested that journalists tend to select sources that are closer to their personal view, and avoid citing those sources that are not in line with their perspective [59]. Therefore, we are confident that by advancing our knowledge of sourcing patterns, this analysis will be helpful in confronting the challenges posed by an era of antivaccine lobbying.

## Figures and Tables

**Table 1:** Main features of the sample.

**Journalistic Genres**	***n***	**%**
News	57	43.5
Feature	29	22.1
Short news	23	17.6
Opinion	15	11.5
Interview	4	3.1
Obituary	1	0.8
Biography	2	1.5
Total	131	100.0
**Tone of the Article**		
Positive	58	44.3
Neutral	55	42.0
Negative	18	13.7
Total	131	100.0
**Type of Vaccine**		
Ebola	13	9.9
Chickenpox	12	9.2
Diphtheria; Meningitis	8	6.1
Influenza; Malaria	7	5.3
Cancer; Zika; Measles	6	4.6
Tuberculosis; HIV	5	3.8
Smallpox	4	3.1
Hepatitis; Whooping cough; Human Papillomavirus; Polio	3	2.3
Pneumococcus	2	1.5
Alzheimer disease; Autism; Dengue; Yellow fever; Gonorrhea; Mumps	1	0.8
General/No identified	24	18.3
Total	131	100
**Frames**		
Human interest	69	52.7
Conflict	43	32.8
Responsibility	9	6.9
Economic	6	4.6
Morality	4	3.1
Total	131	100.0
**Length (Number of Words)**		
1–100	15	11.4
101–200	10	0.8
201–300	12	9.2
301–400	19	15.5
401–500	17	13.0
501–600	12	9.2
601–700	12	9.2
701–800	13	9.9
801–900	11	8.4
901	10	0.8
Min	32	
Max	2158	
Mean	377.7	
Median	480	
Standard deviation	332.0	

**Table 2:** Frequency counts for sources.

**Types of Sources**	***n***	**%**
Government scientific organisations	95	25.4
Professional associations	62	16.5
Government organisations	57	15.2
Scientific companies	39	10.4
University scientists	39	10.4
Scientific journals	30	8.0
Clinicians	25	6.6
NGOs	15	4.0
Media	6	1.6
Consumer groups	3	0.8
Others	3	0.8
Total	374	100
*X^2^* (*df* = 8) = 1.27, *p* = 0.996		
**Category of Sources**	***n***	**%**
Scientific sources	290	77.5
Non-scientific sources	84	22.5
*X^2^* (*df* = 1) = 0.05 *p* = 0.830		
**Number of Sources Per Article**	***n***	**%**
0	12	9.2
1	30	22.9
2	25	19.1
3	22	16.8
4	15	11.5
5	10	7.6
6	9	6.9
7	4	3.1
8	2	1.5
10	2	1.5
Total	131	100.0
*X^2^* (*df* = 9) = 66.18, *p* < 0.001		

**Table 3:** Correlations among source types and the article characteristics (tone, journalistic genre, vaccine type and frame).

Articles Characteristics	Government Scientific Agencies ^1^	Government Organisations ^2^	Scientific Companies ^1^	University Scientists ^1^	Clinicians ^1^	Scientific Journal ^1^	Media ^2^	Professional Associations ^1^	NGO’s ^2^	Scientific Sources	Non-Scientific Sources
Tone	0.49	−0.17	0.08	−0.16	−0.10	−0.31	−0.61	0.36 *	0.05	0.17	−0.07
Journalistic genre	0.04	−0.24	−0.14	0.00	−0.20	0.40 *	--	−0.23	−0.07	−0.37 ***	−0.20 *
Vaccine type	−0.09	−0.01	0.27	−0.07	−0.13	0.03	0.00	−0.45 *	−0.11	−0.18 *	−0.15
Frame	−0.16	0.09	0.70 ***	0.18	−0.05	0.58 **	−0.56	−0.19	−0.42	−0.21 *	0.16
Length of article	0.23	−0.13	0.02	0.26	0.00	0.17	−0.71	0.17	0.37	0.52 ***	0.28 **

Notes: table shows Spearman rho correlations (two-tailed). ***, ** and * indicate statistically significant at the 0.001, 0.01 and 0.05 levels. The sources “patients’ associations” and “others” were not included in the above analysis due to their low frequency (*n* = 3 each). ^1^ Scientific sources category. ^2^ Non-scientific sources category.
